# Simultaneous measurement of biochemical phenotypes and gene expression in single cells

**DOI:** 10.1093/nar/gkaa240

**Published:** 2020-04-14

**Authors:** Amanda L Richer, Kent A Riemondy, Lakotah Hardie, Jay R Hesselberth

**Affiliations:** 1 Department of Biochemistry and Molecular Genetics, Aurora, CO 80045, USA; 2 Molecular Biology Program; 3 RNA Bioscience Initiative, University of Colorado School of Medicine, Aurora, CO 80045, USA

## Abstract

Methods to measure heterogeneity among cells are rapidly transforming our understanding of biology but are currently limited to molecular abundance measurements. We developed an approach to simultaneously measure biochemical activities and mRNA abundance in single cells to understand the heterogeneity of DNA repair activities across thousands of human lymphocytes, identifying known and novel cell-type-specific DNA repair phenotypes.

## INTRODUCTION

New methods to study heterogeneity at cellular resolution measure differences in gene expression (1–4), chromatin accessibility (5) and protein levels (6) across thousands to millions of cells to understand developmental trajectories of tissues, tumors and whole organisms. But these methods only measure static levels of DNA, RNA and proteins, limiting our ability to extract dynamic information from individual cells.

We developed a functional assay as a new modality for single-cell experiments. Our key innovation is that, instead of measuring the abundance of molecules—i.e. levels of DNA, RNA or protein—from single cells and predicting functional states, we directly measure enzymatic activities present in single cells by analyzing the conversion of substrates to intermediates and products in single-cell extracts within a high-throughput DNA sequencing experiment. Our approach is compatible with existing platforms that measure gene expression at single-cell resolution and can measure many different enzymatic activities simultaneously, querying different biochemical activities by combining unique substrates.

We measured DNA repair activities in single cells because the enzymatic substrate (i.e. a DNA lesion to be repaired by cellular enzymes) yields a product that can be directly analyzed by DNA sequencing. DNA damage is repaired by multiple different and often redundant pathways including base excision repair, nucleotide excision repair, mismatch repair and direct reversal (7). Current methods to study DNA repair in cells and cell extracts use synthetic DNA substrates to measure repair activities (8,9), but these approaches do not scale to multiple measurements (i.e. gene expression and biochemical activities) from the same cell, and their reliance on substrate transfection precludes facile application to primary cells.

## MATERIALS AND METHODS

### DNA repair substrates for single cell experiments

Oligonucleotides were purchased from IDT (Supplementary Table S5). Substrates contain a 5′ and 3′ C3 spacer to prevent exonuclease degradation and reverse transcriptase extension of the substrates. Hairpins were gel purified prior to use in single cell experiments. Briefly, 2–5 nmol of hairpins were loaded in denaturing buffer (47.5% formamide, 0.05% Orange G) on 8% 19:1 acrylamide (BioRad) TBE-Urea gels (7 M urea, 0.1 M Tris base, 0.1 M boric acid, 2 mM EDTA). Hairpins were visualized with UV shadowing on a TLC Silica gel 60 F_254_ plate (Millipore), cut from the gel, crushed in a 1.5 ml Eppendorf tube and eluted in 400 μl 0.3 M sodium acetate overnight at 37°C shaking at 400 RPM. Acrylamide was removed using 0.45 μm cellulose acetate filters (Costar). Hairpins were then purified via ethanol precipitation and resuspended in water. The concentration of purified hairpins was determined via absorbance at 260 nm on a Nanodrop 2000 (Thermo Scientific).

### Preparation of single cell suspensions

Single cell suspensions from cell lines were prepared according to 10× Genomics guidelines. Briefly, cells were quickly washed with 0.25% trypsin (ThermoFisher) and then incubated in 0.25% trypsin for 5 min at 37°C. Trypsin digestion was quenched by the addition of cell culture medium. Cells were isolated by centrifugation at 150 × g for 3 min (these same conditions were used for all cell washes). For cell mixing experiments, approximately 10^6^ cells from each knockout cell line (UNG^KO^ or RNASEH2C^KO^) were filtered through a 30 μm strainer and mixed in the same tube. Cells were washed twice with cold PBS containing 0.04% BSA. Cells were resuspended in 500 μl PBS with 0.04% BSA and filtered through a Flowmi™ Tip Strainer. Cells were stained with trypan blue and counted on a hemocytometer. Cell concentration ranged from 400 to 1000 cells per μl and viability was between 80% and 95%.

Fresh peripheral blood mononuclear cells (PBMC) were isolated from whole blood donated by healthy human donors according to University of Colorado IRB guidelines in sodium heparin tubes. Approximately 5–10 ml of whole blood was diluted with PBS to a total volume of 35 ml. Diluted whole blood was layered over 10 ml Ficoll-Paque PLUS (GE) and centrifuged at 740 × g for 20 min with no deceleration. Cells located above the Ficoll layer were removed and washed twice with PBS. Cells were counted and approximately 2 million cells were washed an additional two times with PBS plus 0.04% BSA. Cells were resuspended in 500 μl PBS plus 0.04% BSA and run through a Flowmi™ Tip Strainer. Cells were counted on a hemocytometer: cell concentration ranged from 400–1000 cells per μl and viability was between 80% and 95%.

### Single cell repair measurements using the 10x Genomics platform

The most current version of this protocol is available at: https://dx.doi.org/10.17504/protocols.io.uhyet7w.

Cells were loaded onto the 10× Genomics single cell 3′ expression kit V2 according to the manufacturer's instructions (CG 000075 Rev C) with the following exceptions:

- When preparing the single cell master mix, 5 μl was subtracted from the appropriate volume of nuclease-free water. After the nuclease-free water was added to the master mix and prior to the addition of the single cell suspension, 5 μl of mixed DNA repair substrates were added (see Supplementary Table S6 for substrate concentrations for each experiment).
- The GEM-RT incubation was changed to the following:
  - Lid temperature: 53°C
  - 37°C for 60 min (unless otherwise noted in experiment Supplementary Figure S6)
  - 53°C for 45 min
  - 4°C Hold and proceed directly to GEM-RT cleanup
- After GEM-RT cleanup, DNA repair substrates and products were separated from mRNA prior to cDNA amplification. 0.6× volume of AmpureXP was added to the eluted RT products (21 μl AmpureXP to 35 μl RT product) and incubated for 5 min at room temperature. The sample was placed on a magnetic strip (High on 10× Magnetic Separator) until the liquid was clear. The supernatant was transferred to a new tube since it contained the DNA repair substrates and products. The beads containing the RT products were washed twice with 150 μl of 80% ethanol, then dried for 2 min at room temperature, and eluted in 35.5 μl of Elution Solution 1 according to 10× SPRIselect cleanup protocols. This fraction was used to prepare the mRNA expression library according to manufacturer's instructions. The supernatant was cleaned up by added 1.8× of the original volume of AmpureXP (42 μl), mixed, and then incubated for 5 min at room temperature. The sample was placed on a magnetic strip until the liquid was clear. The supernatant was discarded and the beads were washed twice with 150 μl 80% ethanol. The beads were dried at room temperature for 2 min, then eluted in 20 μl water. This fraction was used to prepare the DNA repair libraries. Note: DNA repair substrates *may* be visible on Tapestation or Bioanalyzer prior to or following size separation, however, this was not measured in these experiments.

### Preparation of DNA repair libraries from single cells

The DNA repair libraries were prepared with the following steps:

1. *End repair*: 20 μl of the purified DNA repair libraries were added to an end repair reaction with a total volume of 30 μl (NEBNext End repair Module E6050) and incubated for 30 min at 20°C.
2. *Clean up by precipitation*: 120 μl of 0.3 M sodium acetate and 400 μl 100% ethanol were added to the end repair reaction (step 1). The reaction was allowed to precipitate at −20°C for at least 30 minutes. Samples were centrifuged at 10 000 × g for 10 minutes and the supernatant was removed and the pellet was washed with 500 μl of 70% ethanol and centrifuged at >10 000 × g for 10 min. The supernatant was removed and the pellet was dried for 2 min at room temperature. The pellet was resuspended in 20 μl nuclease-free water.
3. *A-tailing:* 15 μl of the end repaired DNA repair library was added to an A-tailing reaction with a total volume of 20 μl (1× Blue Buffer (Enzymatics), 400 μM dATP, 5 units Klenow 3′-5′ exo- (Enzymatics)) for 30 min at 37°C. The A-tailing reaction was cleaned up using precipitation as in step 2.
4. *Adapter ligation*: 13 μl of the A-tailing reaction was added to an Illumina Y adapter ligation reaction with a total volume of 20 μl (66 mM Tris–HCl, 10 mM MgCl_2_, 1 mM DTT, 1 mM ATP, 7.5% PEG 6000, pH 7.6, 0.3 μM annealed Y adapters, 600 units Rapid T4 DNA Ligase (Enzymatics)) and incubated at 25°C for 30 min. The ligation reaction was purified using 1.8× volume of Agencourt AMPure XP (Beckman Coulter) beads as described by the manufacturer. The reaction was eluted in 20 μl nuclease-free water.
5. *Illumina TruSeq PCR*: 13 μl of the purified ligation reaction was added to a PCR reaction with a total volume of 50 μl (1× Phusion HF buffer (NEB), 200 μM dNTPs, 0.6 μM ILMN PCR primers (F and R), 2 units Phusion High Fidelity DNA polymerase). 14–20 cycles of PCR were done with 98°C melting temperature for 15 s, 65°C annealing temperature for 15 s and 72°C extension temperature for 15 s.
6. *PCR cleanup and sequencing:* The DNA repair library was purified using 1.2× volume Agencourt AMPure XP (Beckman Coulter) beads as described by the manufacturer. The DNA repair library was quantified using the Qubit HS dsDNA fluorometric quantitation kit (Thermo Scientific). 1 μl of the DNA repair library was analyzed on the Agilent D1000 Tapestation. The DNA repair library was ∼230–250 base pairs (Supplementary Figure S2). The DNA repair library was paired end sequenced on a NovaSeq 6000 system with 2 × 150 base pair read lengths at the University of Colorado Anschutz Medical Campus Genomics and Microarray core. Each library was sequenced with at least 10 million reads per sample.

### Single cell data processing

Data processing scripts are available at https://github.com/hesselberthlab/sc-haircut. Briefly, FASTQ files from the 10× mRNA libraries were processed using the cellranger count pipeline (v3.0.2). Reads were aligned to the GRCh38 reference. For the repair libraries, the cell barcodes and UMIs were extracted from R1 using umi_tools (10). All of the known 10x cell barcodes were provided as the whitelist. R2 was trimmed to remove the 3′ polyA sequence and the 5′ template switching sequence. Then R2 was aligned to a hairpin reference fasta file using bowtie2 (v2.3.2) (11), no reverse complement alignment was allowed to ensure sequences aligned in the correct orientation to the reference. The chromosome (same as substrate name) and 5′ alignment position were concatenated and added to the bam file in the XT flag. UMIs were grouped and appended to the BAM files as a tag using umi_tools group. UMIs were counted per cell per hairpin position using umi_tools count. The table output was converted into a sparse matrix and filtered by matching cell barcodes found in the cellranger filtered feature matrix output using functions in the scrunchy R package (https://github.com/hesselberthlab/scrunchy).

#### Seurat

**Figure 1.**
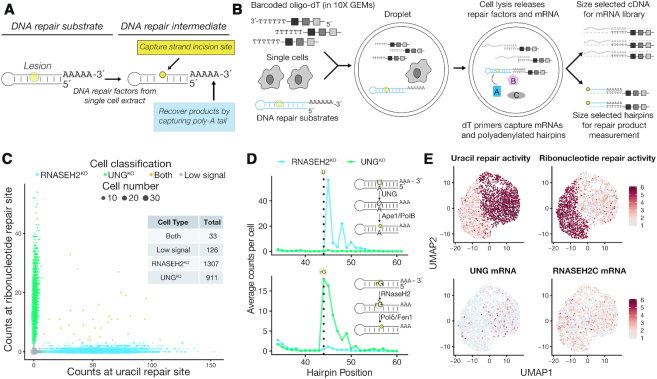
Development and validation of a single-cell assay for measuring DNA repair capacity. (**A**) Schematic of DNA repair substrates used in single-cell Haircut. Strand incision generates a new 5′-end whose position is captured by cDNA synthesis with barcoded oligonucleotides. (**B**) Overview of single-cell Haircut. After droplet formation, cell lysis creates a ∼50 pl reaction wherein enzymes contributed by the cell catalyze substrate turnover (i.e. strand incision for specific DNA repair substrates). Repair products and mRNAs are converted to cDNA with barcoded oligo-dT primers and separated by size to enable separate library preparations. The cDNA for each fraction is analyzed to identify the cell barcode and either mRNA abundance or the amount of enzymatic activity (i.e., number of strand incisions). (**C**) Polyadenylated hairpins containing a single uracil or ribonucleotide (25 nM each) were added to a single-cell suspension of a mixture of Hap1 cells containing null alleles of either UNG or RNASEH2C prior to capture in a 10x Genomics 3′ Gene Expression experiment. Sequences from the DNA repair fraction in (B) were grouped based on their cell barcodes, and the level of strand incision for uracil and ribonucleotide substrates was used to classify cells as either UNG^KO^ (green) or RNASEH2C^KO^ (blue) based on strand incision activity (UMI counts at position 44 for ribonucleotide, position 45 for uracil) >5% of the maximum for all cells. Cells that fall on the x- or y-axis are single RNASEH2C^KO^ and UNG^KO^ cells, respectively; doublets are in yellow; and cells with low signal (<5% of the max for both activities) are in grey. (**D**) Aggregate counts of strand incision events are plotted against hairpin position for cells classified in (C) on the U:A substrate (top) and rG:C substrate (bottom). The vertical dashed line notes the position of the uracil and ribonucleotide (position 44 in both cases). UNG^KO^ cells fail to incise uracil-containing hairpins (green line in top panel) and RNASEH2C^KO^ cells fail to incise ribonucleotide-containing hairpins (blue line in bottom panel). The predominant signal at position 45 (top) reflects UNG conversion of the uracil (position 44) to an abasic site, followed by removal of the abasic site by Ape-1 or Pol β. The predominant signal at position 44 (bottom) reflects 5′-incision of the ribonucleotide at position 44 followed by copying of the rG template by reverse transcriptase in the droplet. Additional processing of incised repair intermediates yields signals at positions 3′ of the lesion (signals at positions 46–50 in the uracil substrate (top), and positions 45–50 in the ribonucleotide substrate (bottom)). (**E**) Variable mRNA expression from cells classified in (C) was used to calculate a UMAP projection (identical coordinates in all four panels). Uracil repair activity (natural logarithm of strand incision counts (position 45 for uracil substrate and position 44 for ribonucleotide substrate; (D) divided by total counts for that cell multiplied by a scaling factor of 10^4^; top left) and ribonucleotide repair activity (top right) were superimposed in a gray-to-red scale and delineate two major cell types in the experiment; 90% of cells in each class have a scored activity. Levels of UNG and RNASEH2C mRNA (natural logarithm of mRNA counts divided by total counts for that cell multiplied by a scaling factor of 10^4^) are plotted on the bottom panels are not sufficient to classify cell types. Stabilization of the null-mutation-containing RNASEH2C mRNA yields uniform RNASEH2C mRNA detection across both cell types.

Downstream analysis of RNA and repair data was performed using the Seurat R package (v3.0.0) (12). Raw, filtered counts for repair was added to the same Seurat object as gene expression. Gene expression counts and repair counts were log normalized (LogNormalize) where feature counts for each cell are divided by the total counts for that cell and multiplied by a scaling factor (10^4^) and then natural-log transformed. PBMC samples were filtered for number of genes per cell >150–200 and <2000–2500 and for percent mitochondrial reads <15–25%. Gene expression data was scaled and centered (ScaleData). 5000 variable features (FindVariableFeatures) were used for PCA calculation (RunPCA) and the first 10–20 principal components were used to find clusters (FindNeighbors, FindClusters) and calculate uniform manifold approximation and projection (UMAP) (RunUMAP). Cell types were identified using the Seurat functions FindTransferAnchors and TransferData (12). Reference PBMC data was downloaded from Seurat vignette and used as reference for PBMC cell types (https://satijalab.org/seurat/v3.1/pbmc3k_tutorial.html; https://support.10xgenomics.com/single-cell-gene-expression/datasets/1.1.0/pbmc3k). Cells were filtered to exclude platelets unless otherwise noted (Supplementary Figure S10). Significant differences and fold changes in repair activities and gene expression between cell types were calculated using Wilcoxon Rank Sum test (FindMarkers, FindAllMarkers) for all pairwise combinations (Supplementary Tables S1, S3 and S4). PBMC replicates were merged and integrated using Seurat functions (FindIntegrationAnchors and IntegrateData) and then analyzed the same as individual replicates. In the cell mixing experiment (Figure 1), cell types were determined by repair activities. Knockout cells were identified if counts at the repair site (position 44 for ribonucleotide and position 45 for uracil) for one repair activity was >5% of the maximum for the repair activity and the other was < 5% of the maximum. If both repair activity counts were >5% of the maximum, that cell was considered a doublet and if both repair activity counts were <5% of the maximum that cell was not classified. Filtered single cell gene expression matrices from previously published data (4) for Supplementary Table S4 and Figure S9 were downloaded from 10x Genomics (https://support.10xgenomics.com/single-cell-gene-expression/datasets/3.0.2/5k_pbmc_v3_nextgem, https://support.10xgenomics.com/single-cell-gene-expression/datasets/3.1.0/5k_pbmc_protein_v3, https://support.10xgenomics.com/single-cell-gene-expression/datasets/3.0.0/pbmc_10k_v3, https://support.10xgenomics.com/single-cell-gene-expression/datasets/2.1.0/pbmc4k) and analyzed the same as above.

#### Genome coverage

To calculate genome coverage for cell mixing experiment (Supplementary Figure S5), BAM files produced by cellranger were split into cell type (as assigned above and in Figure 1) specific BAM files by cell barcodes using samtools view (v1.9) (13). Bulk genome coverage was calculated for UNG^KO^ or RNASEH2C^KO^ cells using bedtools genomecov (v2.26.0) (14). Coverage was visualized with the UCSC Genome Browser (15).

#### Cell type classification with repair data

**Figure 2.**
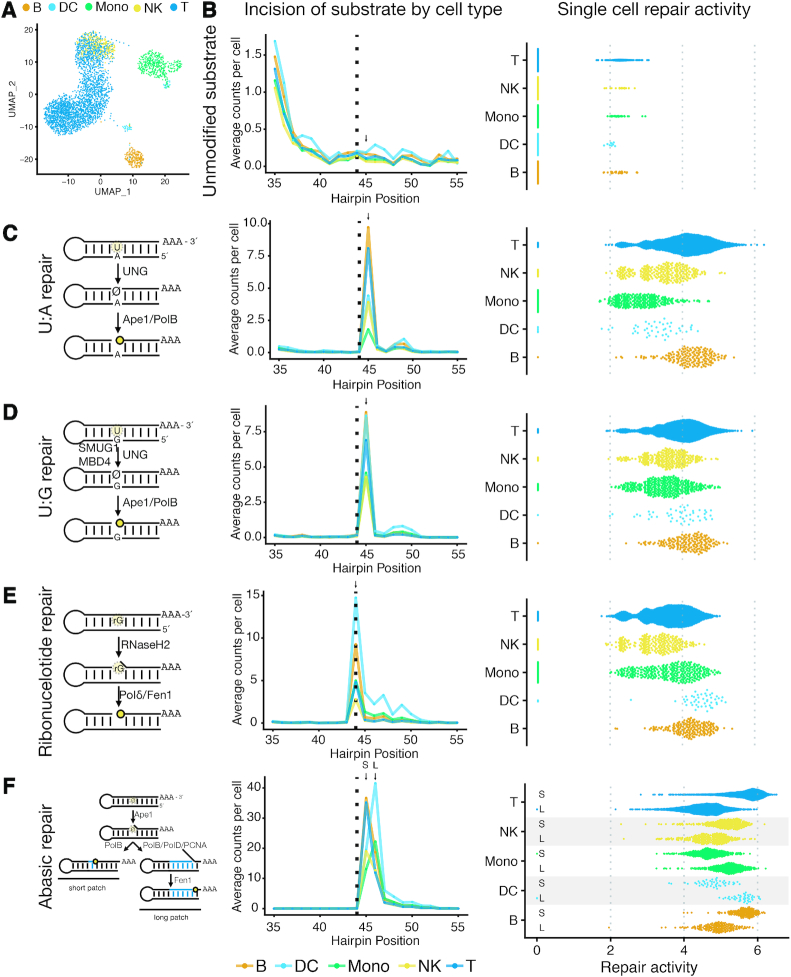
Analysis of DNA repair heterogeneity in human lymphocytes. (**A**) Two-dimensional UMAP projection of variable gene expression across 3,298 human PBMCs captured in a 10× Genomics 3′ Gene Expression experiment. Major cell types were determined by marker gene expression (CD19 for B cells; IL7R for T cells; LYZ, FCGR3A, CD14 for monocytes; FCER1A for dendritic cells; GNLY for NK cells). (**B**) Single-cell DNA repair activity of an unmodified DNA substrate. The dashed vertical line indicates the position of lesions in other DNA repair substrates (left). Unmodified DNA substrates yield few measured incisions in single-cell Haircut with an average count per cell per position of <1. Very few cells have measured repair activity (natural logarithm of strand incision counts (at position 45; arrow, left) divided by total counts for that cell multiplied by a scaling factor of 10^4^), indicating they are not substrates for cellular DNA repair activities. (**C**) Repair of a substrates containing a uracil:adenine (U:A) base-pair initiates with UNG-mediated removal of the uracil nucleobase followed by processing of the abasic site by Ape-1 and Pol β (left). Cell-type-specific counts of incision and processing (mean) are plotted against the position of the hairpin and colored as in (A) (middle). Single-cell repair activities (natural logarithm of counts at the incision site (arrow) divided by total counts for that cell multiplied by a scaling factor of 10^4^) are plotted for each cell from each cell type (right). Monocytes and dendritic cells have reduced uracil incision relative to other cell types (*P* < 10^−140^ monocytes to T cells, and *P* < 10^−8^ dendritic cells to T cells; Wilcoxon signed-rank test; differences significant across 3 replicates and integration of 3 replicates, Supplementary Table S1). (**D**) Repair of a substrates containing a uracil:guanine base-pair initiates with UNG, SMUG, or MBD4-mediated removal of the uracil nucleobase followed by processing of the abasic site by Ape-1 and Pol β (left). Cell-type-specific incisions are plotted as in (**c**) with a predominant incision site one base downstream of the uracil (arrow, position 45), similar to the U:A substrate (A) (middle). The higher uracil repair activity (as defined in (C)) on the U:G relative to U:A substrates for monocytes and dendritic cells likely reflects recognition of the U:G substrate by SMUG and MBD4. (**E**) Repair of a substrates containing a riboG:C base-pair initiates with RNASEH2-mediated incision 5′ of the ribonucleotide followed by processing by Pol δ and Fen1 (left). Cell-type-specific counts of incision and processing (mean) are plotted against the position of the hairpin and colored as in (A) (middle), with the predominant signal at the ribonucleotide, reflecting incision by RNASEH2 and cDNA synthesis using the ribonucleotide template by reverse transcriptase in the droplet (arrow, position 44). B cells and dendritic cells have higher levels of ribonucleotide repair activity (as defined in (C)) than other cell types (*P* < 10^−33^ B cells to T cells; *P* < 10^−15^ dendritic cells to T cells; Wilcoxon signed-rank test, differences significant across three replicates and integration of all three replicates, Supplementary Table S1). (**F**) Repair of substrates containing a abasic:guanine base-pair initiates with Ape-1-mediated incision followed by processing of the single-base gap by either Pol β (short-patch repair) or Pol δ/β and Fen1 (long-patch repair) (left). Cell-type-specific incisions are plotted as in (**c**) with a predominant incision sites one or more bases downstream of the abasic, depending on the cell type (middle). For each cell type, the levels of short-patch (top; signals 1 nt downstream of lesion, S arrow, position 45) and long-patch (bottom; signal 2 nt downstream lesion, L arrow, position 46) repair activities (as defined in (C)) are plotted (right). Monocytes and dendritic cells have lower levels of short-patch repair relative to other cell types (*P* < 10^−162^ monocytes to T cells; *P* < 10^−19^ dendritic cells to T cells; Wilcoxon signed-rank test, differences significant across three replicates and integration of all three replicates, Supplementary Table S1).

To determine whether DNA repair activities are useful in classifying PBMC cell types, we used the PBMC replicates 1 and 2 (Figure 2 and Supplementary Figure S10, left). True cell types were determined using reference PBMC data from 10× Genomics as described in Seurat analysis section above. All other cell type classifications were compared to these reference cell types. Next, cell types were determined by renaming the defined Seurat clusters as the majority cell type present within each cluster (Supplementary Figure S10B). Additionally, mRNA (Supplementary Figure S10c) and/or repair data (Supplementary Figure S10D, E) from PBMC replicate 1 was used as the reference input for assigning cell types to PBMC replicate 2 using Seurat's FindTransferAnchors and TransferData functions. Cell types were randomly reassigned using R (sample) (Supplementary Figure S10F). True positive, true negative, false positive, and false negative numbers were calculated for each cell type for each classification method. These numbers were then used to calculate true positive rate and false positive rate for each cell type for each classification method (Supplementary Figure S10g).

#### Bulk RNA-seq from tissues analysis

Data was downloaded from the Human Protein Atlas (https://www.proteinatlas.org/download/rna_tissue_consensus.tsv.zip) for the RNA consensus tissue gene data set. These data are gene transcript expression levels from 74 tissues. The normalized expression values in the data are the maximum expression value from 3 different sources. These data were visualized using the R package ggplot2 (Supplementary Figure S9).

#### Empty drops vs cells analysis

To determine biological versus background activity in single cells, we calculated hairpin coverage in empty drops (Supplementary Figure S3). Empty drops were determined by filtering out cell-associated barcodes from the repair matrix. The resulting repair matrix contains many barcodes that are associated with only a single UMI, so the matrix was filtered by descending UMI counts to the same number as cell-associated barcodes. This repair matrix from empty drops was used as the input to calculate empty drop signal across the hairpin by calculating the mean count across all drops at each hairpin position.

#### Haircut signal detection sensitivity

To determine the lower limit of haircut signal detection suitable for classifying cells as either UNG^KO^ or RNASEH2C^KO^ read alignments were randomly downsampled using samtools view (v1.9) (13). The downsampled BAM files were then processed using the haircut single cell processing pipeline to produce haircut signal matrices. Cells were classified as either UNG^KO^, RNASEH2C^KO^, doublets, or low signal using the same cutoffs described in the Seurat analysis methods.

#### Estimated recovery of substrates

To determine the proportion of substrates recovered from each droplet (Supplementary Figure S7), the total number of hairpins added to each drop was estimated using the following assumptions:

1. When the oil-water emulsion droplet is formed within the 10× Genomics Chromium chip, the volume of the master mix is doubled, thus reducing the concentration of hairpins to $\frac{1}{2}$ the concentration of hairpins in the master mix.
2. Diameter of each drop: ∼80 μm
3. Volume of each drop: ∼270 pl

The number of substrates in each drop was then calculated using the following formula:

$$\begin{eqnarray*}&&\frac{{{\rm Conc}\ {\rm of}\ {\rm hairpin}\ {\rm in}\ {\rm master}\ {\rm mix}(M)}}{2} \nonumber \\ && \times {\rm Volume}\ {\rm of}\ {\rm drop}(L) \times 6.02 \times\ {10^{23}}\end{eqnarray*}$$

This resulted in the following number of hairpins per drop by concentration in master mix:

**Table:** 

Concentration in master mix (nM)	Approximate number of hairpin molecules in each drop
0.5	40 000
2.5	200 000
5	400 000
10	800 000
25	2 000 000

To determine the proportion of hairpins recovered per drop, the total number of aligned reads per hairpin per drop was divided by the approximate number of hairpins per drop.

#### Direct reversal substrate and 5′ biotin cleanup

To measure repair of direct reversal substrates we included O^6^methyl-G substrate in PBMC experiments (Supplementary Figures S3 and S8). Prior to end repair, the repair fraction was digested with PstI (NEB, 20 U in 1× Cutsmart buffer) at 37°C for 60 min. The reaction was cleaned up by precipitation, followed by the above steps starting at end repair. To remove background signal on the 5′ end of the substrates, we included a uracil substrate with a 5′ biotin in PBMC experiments (Supplementary Figures S3 and S8). To remove uncleaved substrate, prior to end repair, the repair fraction was incubated with Dynabeads™ M-270 Streptavidin (5 μg, Thermo) for 5 minutes at room temperature. Following incubation, the beads were discarded and the supernatant was cleaned with precipitation. The remainder of the protocol proceeded starting from end repair.

### Oligonucleotides for repair libraries

Other oligonucleotides used in the library preparation can be found in Supplementary Table S5. To anneal Y adapters, 100 μM Y adapter 1 and 2 were mixed in equimolar concentration in 10 mM Tris–HCl pH 7.5, 50 mM NaCl and heated to 95°C and cooled to 4°C over 5 min. Annealed adapters were diluted to 10 μM final concentration in cold 10 mM Tris–HCl pH 7.5, 50 mM NaCl. Annealed adapters were stored at –20°C until use.

### Cell lines and cell culture

Hap1 UNG^KO^ (HZGHC001531c012) and RNASEH2C^KO^ (HZGHC004633c003) cells were purchased from Horizon Discovery. Cell lines were cultured in IMDM (Gibco, purchased from ThermoFisher) supplemented with 10% FBS (ThermoFisher) and Penicillin-Streptomycin (ThermoFisher) at 37°C with 5% CO_2_.

### RT-qPCR

Total RNA from cells was isolated using TRIzol reagent (ThermoFisher) according to manufacturer's instructions. Total RNA (5 μg) was treated with TURBO DNAse (2 U) (ThermoFisher) according to manufacturer's instructions. Following DNAse treatment, 1 μg of total RNA was reverse transcribed using Superscript II (200 U, ThermoFisher) and random hexamers primers (0.5 μM, ThermoFisher) to make cDNA. The cDNA was then used for quantitative PCR (qPCR) using Sso Advanced Universal SYBR Green Supermix (Bio Rad) and cycled on a Bio Rad C1000 384-well thermal cycler and plate reader. qPCR experiments were done in technical triplicate and biological duplicates.

## RESULTS AND DISCUSSION

We included synthetic DNA hairpins with polyadenylate tails and DNA lesions at defined positions (Figure 1A) in a single-cell mRNA sequencing experiment and developed a protocol to capture incised DNA repair intermediates and products from single-cell extracts by library preparation and sequencing (Figure 1B and Supplementary Figures S1 and S2). Because our method employs massively-parallel measurement of strand incision on DNA hairpins, we named it ‘Haircut’.

Cell mixing experiments confirmed we could measure DNA repair activities at cellular resolution with single-cell Haircut. We added DNA repair substrates with a uracil (U:A base-pair) or ribonucleotide (rG:C) to a single-cell suspension of haploid human UNG^KO^ and RNASEH2C^KO^ knockout cells immediately prior to emulsion formation in a droplet-based single-cell mRNA sequencing experiment (10× Genomics 3′ Gene Expression; Figure 1b). After sequential incubations at 37 and 53°C to first promote endogenous enzymatic activity and then reverse transcription, the emulsion was broken and cDNA molecules synthesized from mRNA and repair substrate templates (the ‘repair fraction’) were separated by size. The mRNA fraction was subjected to the standard protocol to measure gene expression for single cells, whereas the repair fraction (containing DNA substrates, intermediates, and products) was captured in a modified protocol (Supplementary Figures S1 and S2). Analysis of the mRNA fraction by DNA sequencing yielded the mRNA identity, a cell-specific barcode, and a unique molecular identifier (UMI (16)). Similarly, DNA sequencing of the repair fraction yielded the cell barcode, UMI, and a 5′ position derived from cDNA synthesis on either full-length hairpins or incised repair intermediates and products.

We captured thousands of single cells with expected DNA repair defects: RNASEH2C^KO^ cells could incise uracil but not a ribonucleotide repair substrate, and vice versa for UNG^KO^ cells, with a few droplets containing more than one cell from each genotype and therefore both uracil and ribonucleotide repair activities (Figure 1C, D). DNA repair associated incision and processing activities were measured at expected positions based on known repair pathways (Figure 1D, right) and were only present in cell-associated droplets (as determined from mRNA signals; Supplementary Figure S3). We calculated repair activity for each cell as the natural logarithm of strand incision counts at the primary repair site (position 45 for the uracil substrate and position 44 for the ribonucleotide substrate) divided by total counts for that cell multiplied by a scaling factor of 10^4^. We also calculated two-dimensional UMAP projections based on variable mRNA expression and colored individual cells by enzymatic activity (UNG or RNASEH2) and mRNA abundance (Figure 1E). DNA repair activity was robustly detected for each cluster (Figure 1E, top row, Supplementary Figure S4) and was sufficient to assign 75% of cells to the correct cell type using 1500 reads-per-cell (Supplementary Figure S4), similar to read depths required for cell type classification using gene expression (17). While UNG (229 cells) and RNASEH2C (1075 cells) mRNAs were identified in these cells, they were not variably expressed across cell clusters (Supplementary Figure S4). Moreover, our analysis of RNASEH2C mRNA levels in RNASEH2C^KO^ cells found that whereas the mutation yields cells that lack RNASEH2 activity (Figure 1E, top right), it does not cause mRNA decay (Supplementary Figure S5), with similar mRNA isoform abundance detected in both cell types. Altogether, this experiment illustrates the unique and orthogonal information provided by single-cell biochemical assays, which is especially useful in situations where mRNA expression is not predictive or may even be misleading of functional status.

DNA repair activity measurements in single-cell extracts from human peripheral blood mononuclear cells (PBMCs) with separately barcoded uracil and ribonucleotide repair substrates spanning a 50-fold range in concentration showed that measured DNA repair signals change as a function of substrate concentration and time (Supplementary Figure S6). Moreover, the proportion of DNA repair substrates recovered in the assay was independent of substrate, concentration, and incubation time (Supplementary Figure S7). Differences in DNA repair among cell types persisted independent of substrate concentration and time, enabling us to choose a single substrate concentration and time point (10 nM substrates at 60 min) for further experiments (Supplementary Figure S6, Table S1).

We next used single-cell Haircut to measure the biochemical heterogeneity of DNA repair in PBMCs using five DNA substrates (unmodified, and containing U:A, U:G, rG:C and abasic:G lesions - added at 10 nM each; Figure 2, Supplementary Figure S8). We were unable to measure DNA repair activity on several other base excision repair substrates (I:C, I:T, EthenoA:T, hydroxymethyl-U:A) and one direct reversal substrate (O^6^mG:C) (Supplementary Figure S3), either due to the sensitivity of the assay (I:C, I:T or hydroxymethylU:A) or the assay specificity (EthenoA:T and O^6^mG:C). We used single-cell mRNA expression to classify cells based on expression of common cell-type-specific markers (e.g. IL7R for CD4+ T cells, CD14 for monocytes; Figure 2A, Supplementary Figure S8) and then used these classifications to assign cell-type-specific DNA repair activities (Figure 2B–F, Supplementary Figure S8).

We found little signal on the unmodified DNA substrate, confirming it is not a repair substrate. In contrast, incision and processing activities measured on uracil (on U:A and U:G substrates), ribonucleotide, and abasic repair substrates matched expected positions based on known repair pathways (left and middle panels in Figure 2C–F) and were only present in cell-associated droplets (as determined from mRNA signals; Supplementary Figure S3). These data revealed unique signatures of DNA repair activities in specific cell types. Monocytes and dendritic cells had low incision activity on the U:A substrate (Figure 2C, Supplementary Figure S8), resonating with the low level of uracil base excision in monocytes (18) and confirming that myeloid lineages have unique uracil repair phenotypes (19) consistent with lower UNG mRNA expression in these cell populations (Supplementary Figure S9). However, these differences in uracil repair were not apparent for the U:G substrate, presumably due to redundant activities of SMUG1 (20) and MBD4 (21) in incising U:G-containing substrates. Dendritic cells demonstrated a unique repair phenotype, with increased levels of DNA substrate processing, measured as increased signals downstream of the position of the synthetic lesion. To explain these differences in DNA repair phenotypes, we examined cell type-specific mRNA expression and found that expression of APEX1, encoding the abasic endonuclease Ape-1, was consistently and uniquely elevated in dendritic cells (Supplementary Tables S2 and S3), possibly explaining the increased processing of DNA repair intermediates on U:A, U:G and abasic substrates in dendritic cells.

B cells and dendritic cells also had higher levels of ribonucleotide repair activity than other cell types (Figure 2 and Supplementary Figure S8), and the increase in ribonucleotide repair in B cells and dendritic cells was corroborated by elevated expression of some RNASEH2 subunits in our (Supplementary Tables S2 and S3) and previous single-cell mRNA sequencing data sets (Supplementary Table S4 and Figure S9) (4). Increased RNASEH2 activity in B cells may aid processing of R-loops that form during class switching (22).

Finally, we focused on cell-type-specific differences in repair of a DNA hairpin containing a synthetic abasic site. These substrates undergo two unique events in droplets: on intact substrates, reverse transcription halts at the abasic site, yielding extension products that map one base downstream of the abasic site (Figure 2F). Alternatively, incision and removal of the abasic site by Pol β and Fen1 (23) yields repair intermediates with 5′-ends that map further downstream of the abasic site. Differences in signals from the abasic substrate specifically in monocytes and dendritic cells again indicate that they are more proficient at processing abasic lesions, evidenced by an increase in levels of intermediates 2 nt downstream of the abasic site (position 46; Figure 2F and Supplementary Figure S8), likely due to elevated Ape-1 expression. The unique DNA repair phenotype provided some power for cell type classification (Supplementary Figure S10g). As additional activities are multiplexed with DNA repair, we expect the power of single-cell biochemical measurements for cell type classification will increase.

Our approach to measure heterogeneity of single-cell biochemical phenotypes can be expanded to measure other types of DNA repair activities (e.g. nucleotide excision repair and mismatch repair) and adapted to measure other enzyme classes using substrate–DNA conjugates (24), enabling simultaneous measurement of many biochemical activities (e.g. kinases, phosphatases and proteases) with gene expression at single-cell resolution.

## DATA AVAILABILITY

Sequencing data have been deposited at NCBI GEO under accession GSE130117. A full protocol is available at https://dx.doi.org/10.17504/protocols.io.uhyet7w. An analytical and reproducible pipeline is available at https://github.com/hesselberthlab/sc-haircut.

Gene Expression Omnibus GEO: https://www.ncbi.nlm.nih.gov/geo/query/acc.cgi?acc=GSE130117.

## Supplementary Material

gkaa240_Supplemental_Files
