# Supplementary material for: Simultaneous measurement of biochemical phenotypes and gene expression in single cells
Source: Nucleic Acids Res. 2020 Apr 14;48(10):e59. doi: 10.1093/nar/gkaa240 (PMC7261187; doi:10.1093/nar/gkaa240)
Supplement: gkaa240_Supplemental_Files [file gkaa240_supplemental_files.zip › Supplementary Text - revised V2.docx]

## **SUPPLEMENTAL TABLES**

### **TABLE 1. Differential repair calculated for biological repair positions**

Significant differences and fold changes in repair activities between cell types and all other cells and all pairwise comparisons were calculated using Wilcoxon Rank Sum test (FindAllMarkers, FindMarkers, Seurat v3.0.0 [(21)](https://paperpile.com/c/sp0tIn/Cmxb)) for all cell types. Statistics were calculated for each replicate individually (sample: pbmc1, pbmc2, pbmc3) and for all samples combined (sample: all_cells). Statistics were also calculated for time course experiment where droplets were incubated for 15, 30, or 60 minutes at 37 °C prior to reverse transcription (**Supplementary Fig. 6**) (15min, 30min, 60min). The result was filtered for adjusted *P* < 0.05 and repair positions (**Fig. 2**). Column information is as follows (from Seurat documentation for FindMarkers):

- gene: concatenation of repair substrate and position. Uracil slope and riboG slope are calculated as in **Supplementary Fig. 6**.
- p_val: p value of Wilcoxon Rank Sum test
- avg_logFC: The log fold-change of average repair activity between group and all other cells. Positive values indicate that the repair activity is greater in the first group.
- pct.1: Percentage of cells where the repair activity was detected in the group.
- pct.2: Percentage of all other cells where the repair activity was detected.
- p_val_adj: Adjusted p-value, based on bonferroni correction using all hairpin positions in the dataset
- sample: The replicate for which the statistics were calculated. “All_cells” is an integrated sample of all replicates. Time course samples are indicated with the 37 °C incubation time.

### **TABLE 2. Differential gene expression.**

Significant differences and fold changes in gene expression between cell types and all other cells were calculated using Wilcoxon Rank Sum test (FindAllMarkers, Seurat v3.0.0 [(21)](https://paperpile.com/c/sp0tIn/Cmxb)) for all cell types. Statistics were calculated for each replicate individually (sample: pbmc1, pbmc2, pbmc3) and for all samples integrated (sample: all cells). Column information is as follows (from Seurat documentation for FindMarkers):

- gene: Gene name
- cluster: Cell type determined by mRNA expression
- p_val: p value of Wilcoxon Rank Sum test
- avg_logFC: The log fold-change of average expression between group and all other cells. Positive values indicate that the gene is more highly expressed in the first group.
- pct.1: Percentage of cells where the gene was detected in the group.
- pct.2: Percentage of all other cells where the gene was detected.
- p_val_adj: Adjusted p-value, based on bonferroni correction using all genes in the dataset
- sample: The replicate for which the statistics were calculated. “All_cells” is an integrated sample of all replicates.

### **TABLE 3. Differential gene expression for base excision repair genes.**

Significant differences and fold changes in gene expression between cell types and all other cells were calculated using Wilcoxon Rank Sum test (using the FindAllMarkers function from Seurat v3.0.0 [(21)](https://paperpile.com/c/sp0tIn/Cmxb)) for all cell types. The result was filtered for genes within the KEGG base excision repair pathway (hsa03410). Statistics were calculated for each replicate individually (sample: pbmc1, pbmc2, pbmc3) and for all samples integrated (sample: all cells). Columns are the same as in **Supplementary Table 2**.

### **TABLE 4. Differential gene expression for base excision repair genes from reference single cell mRNA datasets.**

Significant differences and fold changes in gene expression between cell types and all other cells and all pairwise comparisons were calculated using Wilcoxon Rank Sum test (FindAllMarkers, FindMarkers, Seurat v3.0.0 [(21)](https://paperpile.com/c/sp0tIn/Cmxb)) for all cell types. Samples were downloaded from 10x Genomics [(4)](https://paperpile.com/c/sp0tIn/vjG3). Data from Donors A, B and C (frozen) [(4)](https://paperpile.com/c/sp0tIn/vjG3) were used as well as reference data from 5K PBMC using NextGem technology (<https://support.10xgenomics.com/single-cell-gene-expression/datasets/3.0.2/5k_pbmc_v3_nextgem>), 5K PBMC using V3 chemistry (<https://support.10xgenomics.com/single-cell-gene-expression/datasets/3.1.0/5k_pbmc_protein_v3>), and 10K PBMC using V3 chemistry (<https://support.10xgenomics.com/single-cell-gene-expression/datasets/3.0.0/pbmc_10k_v3>). Results were filtered for adjusted *P* < 0.05 and genes within the KEGG base excision repair pathway (hsa03410). Columns are the same as **Table 2** with the addition of celltype1 and celltype2 indicating the pairwise comparisons.

### **TABLE 5. Oligonucleotides for single cell DNA repair measurements**

Substrates and other oligonucleotides used in single-cell Haircut experiments. We did not measure biological activity from the following substrates in single cells: C:I, T:I, T:ethenoA, C:O6mG, A:hmU (**Supplementary Fig. 3)**. These substrates were included in single-cell Haircut experiments with human PBMCs (**Supplementary Table 6**).

### **TABLE 6. DNA repair substrate experimental conditions**

This table contains the substrates and concentrations used in single-cell Haircut experiments.

## **SUPPLEMENTARY FIGURE LEGENDS**

### **Supplementary Figure 1. Schematic of single-cell Haircut library preparation.**

DNA repair substrates are added to a 10x Genomics Chromium Single Cell 3′ v2 kit. Within each droplet, DNA repair substrates are exposed to cell extract containing endogenous active DNA repair enzymes. Additionally, each drop contains a reverse transcriptase and an oligo-dT reverse transcription primer. Cell-derived DNA repair enzymes initiate DNA repair on the substrates creating a strand incision, oligo-dT primer and reverse transcriptase present in the drop capture the DNA repair intermediates along with cellular mRNAs. After the emulsion is broken, DNA repair substrates are separated from mRNAs by size. The 5′ site of strand incision is captured through end repair, A-tailing, ligation of a TruSeq adapter, followed by PCR. This library is compatible with next generation sequencing.

**Supplementary Figure 2. Quality control of DNA repair libraries.**

**a.** Gel Red stained 10% acrylamide 29:1 Tris-Borate-EDTA gel image (left, prior to Ampure cleanup) and Tapestation D1000 trace (right) of repair library from mixed knockout Hap1 cells lines (**Fig. 1**). Library size is between 200 and 250 base pairs.

**b.** Gel Red stained 10% acrylamide 29:1 Tris-Borate-EDTA gel image (left) and Tapestation D1000 trace (right) of repair library from fresh human PBMCs (**Fig. 2**). Library size is between 200 and 250 base pairs.

### **Supplementary Figure 3. Determining biological DNA repair activity from single-cell Haircut signals.**

**a.** Counts per cell (mean) (orange) by position were compared to counts per empty drop (mean) to determine which positions contained biological activity. Empty drops were determined by filtering out cell-associated barcodes from the unfiltered repair matrix. The resulting repair matrix contains many barcodes that are associated with only a single UMI, so the matrix was filtered by descending UMI counts to the same number of cell-associated barcodes. This repair matrix was used as the input to calculate empty drop signal across the hairpin by calculating the sum across all drops at each hairpin position. Positions exhibiting signal above empty drop background and associated with a known DNA repair position (e.g., position 45 for U:A) were considered biologically relevant. Some signals above background at the 5′ end of the substrate could be due to cellular exonuclease activity and were not considered in the analysis.

**b.** Coverage across all cells (orange) or across cell types (blue, green) and empty drops (grey) for barnyard experiment (**Fig. 1**). While signals at the 5´ end of the substrate in cells are higher than signals in empty drops, only signals at or adjacent to the site of the lesion (dotted line) are consistent with known DNA repair pathways (**Fig. 1**) and are dependent on the presence of DNA repair factors.

**c.** Coverage across all cells (orange) and empty drops (grey) for PBMC experiment (**Fig. 2**). C:I and T:I substrates did not show biological activity above background.

**d.** Coverage across all cells (orange) and empty drops (grey) for PBMC experiments (**Supplementary Fig. 8a**). The Uracil-5′ biotin substrate contained a 5′-biotin. During the library preparation, uncleaved substrate was removed using streptavidin beads. We only saw a modest reduction in signal on the 5′ end of the substrate in cell-containing and empty drops. The hmU substrate did not have signal above empty drop background. The ethenoA substrate did not have signal above background due to a high level of background signal in empty drops, which could be due to the bulky ethenoA lesion causing the RT to stop. To measure direct reversal substrates, we included an O6mG containing hairpin. Following substrate isolation from the mRNA fraction, substrates were digested with PstI to measure the removal of O6mG [(26)](https://paperpile.com/c/sp0tIn/76sx); however, we measured digestion in empty drops as well as drops with cells indicating the method was not specific for droplets containing cells.

### **Supplementary Figure 4. DNA repair measurements determine cell types in a cell mixing experiment.**

**a.** UMAP plot of cell mixing experiment (**Fig. 1)**. mRNA expression was used to calculate UMAP projections and cluster cells. Cells were clustered using an unsupervised shared nearest neighbors method in Seurat (FindNeighbors, FindClusters). Cells were colored by the resulting seven cluster numbers.

**b.** A table of cells expressing UNG mRNA, RNASEH2C mRNA, uracil repair activity, or ribonucleotide repair activity in each cluster. UNG mRNA was detected in < 10% of cells while RNASEH2C mRNA was detected in ~45% of cells. One or both repair activities were measured in >90% of cells.

**c.** Beeswarm plots of UNG mRNA expression, RNASEH2C mRNA expression, uracil repair activity, or ribonucleotide repair activity by cluster. To determine if UNG and RNASEH2C mRNA expression could be used to assign cell types, we made beeswarm plots of UNG expression and RNASEH2C expression and attempted to assign cell types based on expression levels by cluster (left 2 plots). Due to few cells with UNG or RNASEH2C mRNA measurements we could not assign cell types independent of repair measurements. To assign cell types by repair activity, we made beeswarm plots of repair activities by clusters (right 2 plots). Clusters with high uracil repair activity and low ribonucleotide repair activity were assigned as RNASEH2C^KO^ cells (clusters 0, 1, and 5). Clusters with high ribonucleotide repair activity and low uracil repair activity were assigned as UNG^KO^ (clusters 2, 3, and 4). Cluster 6 was was assigned as having both repair activities.

**d.** Counts at ribonucleotide repair site and uracil repair site were plotted and colored by cluster (left), cell type as determined above (**c**) (middle), and cell types as determined in **Fig. 1** (right). Cell types as determined in **Fig. 1** show the greatest segregation in these plots.

**e.** BAM files from the cell mixing experiment in **Fig. 1** were down sampled to different sequencing depths to determine how many reads per cell are required for cell type classification. At each threshold, cell types were classified as in **Fig. 1** and the percent of cells that can be classified as either UNG^KO^ or RNASEH2C^KO^ is plotted as a function of sequencing depth. Approximately 75% of the 2,377 cells are correctly classified at 1,500 reads per cell.

### **Supplementary Figure 5. Single-cell mRNA expression of RNASEH2C and UNG in Hap1 knockout cells.**

**a.** Protein domains for RNASEH2C (top) and mutant RNASEH2C (bottom) from Horizon knockout line. A 10 base pair deletion in the RNASEH2C^KO^ Hap1 line causes a frameshift at amino acid 19 that leads to 60 amino acid substitutions and a stop codon at position 79, disrupting the heterotrimeric interaction regions of RNASEH2C [(25)](https://paperpile.com/c/sp0tIn/8hA6).

**b.** mRNA coverage of RNASEH2C from single-cell RNA sequencing data (**Fig. 1**). Coverage from all cells from negative strand is in orange. Cell types were identified by repair of uracil-containing or ribonucleotide-containing substrates (**Fig. 1**). Alignment files were then separated by cell barcodes associated with cell types. Bulk mRNA coverage for RNASEH2C for RNAESH2C^KO^ cells (blue) and UNG^KO^ cells (green) show similar coverage for the RNASEH2C gene independent of cell type. Coverage for the RNASEH2C gene is located near the 3′ end of the 2 mRNA isoforms (gene diagrams on bottom). Horizon RNASEH2C^KO^ sequencing results provided from Horizon Discovery displayed on bottom (RNASEH2C^KO^ mutation). qPCR primers are also displayed on bottom (F1/2, R1/2).

**c.** Protein domains for UNG (top) and mutant UNG (bottom) from Horizon knockout line. An 11 base pair deletion leads to a frameshift at amino acid 88, 49 amino acid substitutions, and a stop codon at amino acid 138 - likely leading to a truncated protein lacking the catalytic domain.

**d.** mRNA coverage of UNG from single-cell RNA sequencing data. Coverage from all cells from positive strand is in orange. Cells were identified by repair of uracil-containing or ribonucleotide-containing substrates. Alignment files were then separated by cell type. mRNA coverage for RNASEH2C^KO^ cells (blue) and UNG^KO^ cells (green) show reduced UNG mRNA coverage in UNG^KO^ cells. Gene diagram is on bottom. Horizon UNG^KO^ sequencing results provided from Horizon Discovery is displayed on bottom (UNG^KO^ mutation). qPCR primers are also displayed on bottom (F1/2, R1/2).

**e.** Quantitative PCR results for RNASEH2C and UNG mRNA confirm single-cell RNA sequencing results. RNASEH2C^KO^ cells express RNASEH2C mRNA. UNG^KO^ cells do not express UNG mRNA at high levels. Error bars represent errors across biological duplicates and 2 sets of primers indicated in **b** and **d**.

### **Supplementary Figure 6. Measuring DNA repair in single cells across multiple concentrations and time points.**

**a.** DNA repair activities were measured in healthy human PBMCs. Uracil (U:A) and ribonucleotide (rG:C) substrates were mixed and added in a range of concentrations (0.5, 2.5, 5, 10, and 25 nM each). After the emulsion was created, the sample was separated into 3 tubes and incubated for 15, 30, or 60 min at 37 ̊C prior to reverse transcription at 53 ̊C. 800-1,500 cells were captured at each timepoint. Cell types were identified using gene expression markers using Seurat and visualized on UMAP projections.

**b.** Repair activities measured increased as substrate concentration increased. Repair activity increased over time from 15 min to 60 minutes. Differences in repair between cell types were consistent across substrate concentrations and most remained significant (*P* < 0.05, **Supplementary Table 1**).

**c.** A linear model (log normalized count at repair site (as defined in **Fig. 2**) ~ substrate concentration) was fitted for each cell for each repair activity. The slope of each linear model is a measurement of repair activity. Repair activity increases as time increases. Additionally, repair activity trends measured using a single concentration and time point are consistent with activity measurements made across substrate concentration (e.g., monocytes have lower uracil repair compared to B cells and T cells, significant *P* values in **Supplementary Table 1**).

**Supplementary Figure 7. Proportion of DNA substrates captured per cell.**

Uracil and ribonucleotide containing DNA repair substrates mixed at 5 concentrations (0.5, 2.5, 5, 10, and 25 nM) and were added to the cells/master mix in a PBMC experiment. After the emulsion was created, the sample was separated into 3 tubes and incubated for 15, 30, or 60 min at 37 ̊C prior to reverse transcription at 53 ̊C. 800-1,500 cells were captured at each timepoint (**Supplementary Fig. 6**). The number of hairpin molecules in each drop was estimated by assuming each drop was ~300 pL in volume and the hairpin concentration in the droplet is half that of the concentration in the master mix. We captured approximately 0.01-0.025% of the hairpins that were added to the assay (with ~10 million total library reads). The proportion of hairpins recovered was independent of the concentration with the exception of the lowest concentration of 0.5 nM and the uracil hairpin at 2.5 nM. The proportion of hairpins recovered was also independent of the 37 ̊C incubation time.

### **Supplementary Figure 8. Biological replication of DNA repair phenotypes in human PBMCs.**

**a-b.** PBMCs were isolated from a single healthy human donor in two batches. 3 ́ single cell gene expression and DNA repair activities were measured for each batch. mRNA expression was used to cluster cells and classify cell types. UMAP plot of cell types (top). Cell-type-specific counts of incision and processing (mean) are plotted against the position of the hairpin (left). Trends for single cell repair of U:A, U:G, riboG:C, and abasic:G substrates were similar across all three replicates (**Fig. 2 and a-b**).

**c.**  Single cell gene expression and repair measurements for 3 replicates (**Fig. 2, and a-b**) were integrated into one Seurat object. mRNA expression was used to cluster cells. Cell types were identified separately for each sample. UMAP plot of cell types and sample number (top) indicate that cells cluster with cell types rather than with samples. Cell-type-specific counts of incision and processing (mean) are plotted against the position of the hairpin (left). Trends for single cell repair of U:A, U:G, riboG:C, and abasic:G substrates were similar to individual replicates (**Fig. 2 and a-b**).

### **Supplementary Figure 9. Gene expression of UNG and RNASEH2 in single cells and cell populations.**

**a.**  Repair of a substrate containing a uracil:adenine (U:A) base-pair initiates with UNG-mediated removal of the uracil nucleobase followed by processing of the abasic site by Ape-1 and Pol β (left). Single-cell repair activities (natural logarithm of counts at the incision site divided by total counts for that cell multiplied by a scaling factor of 10^4^) are plotted for each cell from each cell type (right) (Also in **Fig. 2**).

**b.** UNG mRNA in single cells from our data (left) and data from 10x Genomics (middle) is not robustly detected in single cells at sequencing depths of 8,000 or 87,000 reads per cell. UNG expression from bulk RNA sequencing experiments (right) correlates with repair activities measured in single cells (**a**).

**c.** Repair of a substrates containing a riboG:C base-pair initiates with RNASEH2-mediated incision 5′ of the ribonucleotide followed by processing by Pol δ and Fen1 (left). Single-cell repair activities (natural logarithm of counts at the incision site divided by total counts for that cell multiplied by a scaling factor of 10^4^) are plotted for each cell from each cell type (right) (Also in **Fig. 2**).

**d.** Single cell measurements of RNASEH2 subunit mRNA from our data (left) and data from 10x Genomics (middle) are not robustly detected at sequencing depths of 8,000 or 87,000 reads per cell. RNASEH2 subunit expression from bulk RNA sequencing experiments (right) is difficult to correlate to repair activities measured in single cells (**a**). The catalytic subunit RNASEH2A does not greatly vary between cell types. While the structural subunit RNASEH2B roughly correlates to RNASEH2 repair activity measured in single cells, this phenotype would be difficult to predict given that each subunit is required for enzymatic function.

### **Supplementary Figure 10. Cell-type classification using DNA repair measurements.**

**a.** PBMC UMAP and cell classifications for PBMC replicate 2 (**Supplementary Fig. 8a**). Cells were classified using reference data from 10x Genomics and Seurat v3.0.0 FindTransferAnchors and TransferData functions. These cell classifications are used as the true cell type for other classification methods, however, these classifications may not be 100% accurate. The percentage of each cell type that is classified the same as this reference data (bottom) is 100% since it is compared to itself.

**b.** Seurat clusters (0-10) were renamed for the majority cell type marker from **a** in each cluster. UMAP plot of renamed cells (top). Using this classification method 93% of cells are classified the same as **a**. > 89% of each cell type were classified the same (bottom).

**c.** mRNA data in **Fig. 2** was used as the reference for classifying cell types in PBMC replicate 2 (**Supplementary Fig. 8)** using Seurat as before (**a**). Using this reference, 93% of the cells were classified the same. > 87% of each cell type were classified the same (bottom).

**d.** DNA repair measurements alone from PBMCs (**Fig. 2**) was used as the reference for classifying cell types PBMC2 replicate. Using only DNA repair for reference data, only 43% of cells were classified the same as **a**. Platelets lack DNA repair activities in Haircut (data not shown) which could contribute to their identification using DNA repair measurements alone as they are distinctly different from all other cells. > 90% of T cells were also identified using repair alone, however, T cells make up ~40% of all cells in the data and when classified using repair data alone, ~80% of all cells are classified as T cells, so by chance alone,T cells are more likely to be classified correctly. Dendritic cells make up only 2.6% of all cells in the data, however, 33% of them are classified correctly using repair activity data alone. DC have several unique repair signatures (**Fig. 2** and **Supplementary Fig. 8**) which are likely to contribute to their classification using DNA repair activities alone.

**e.** Count matrices for mRNA expression and DNA repair were combined and used as a reference (from **Fig. 2** data) to classify PBMC replicate 2 cells. Using this reference, 93% of the cells were classified the same as in **a**. > 86% of each cell type were classified the same (bottom).

**f.** Cell type labels from PBMC replicate 2 as defined in **Supplementary Fig. 8** were randomly reassigned to cell ids. Only 31% of cells were classified the same. More abundant cell types were more likely to be correctly classified by chance (bottom). Error bars represent the 95% confidence interval for 1000 independent samplings.

**g.** True positive rate (# of true positives / (# of true positives + # of false negatives)) and false positive rate (# of false positives / (# of false positives + # of true negatives)) for each cell type classified by each method. Cells classified using mRNA expression with or without repair had a high true positive rate and low false positive rate. Most cell types defined by repair alone had relatively equal true and false positive rates, with the exception of platelets which had a high true positive and low false positive rate and DC which had a relatively low true positive rate but a very low false positive rate indicating DNA repair measurements for these cell types may be helpful for classification.
