# Supplementary material for: Simultaneous measurement of biochemical phenotypes and gene expression in single cells
Source: Nucleic Acids Res. 2020 Apr 14;48(10):e59. doi: 10.1093/nar/gkaa240 (PMC7261187; doi:10.1093/nar/gkaa240)

Supplementary Figure 1
Richer et al.

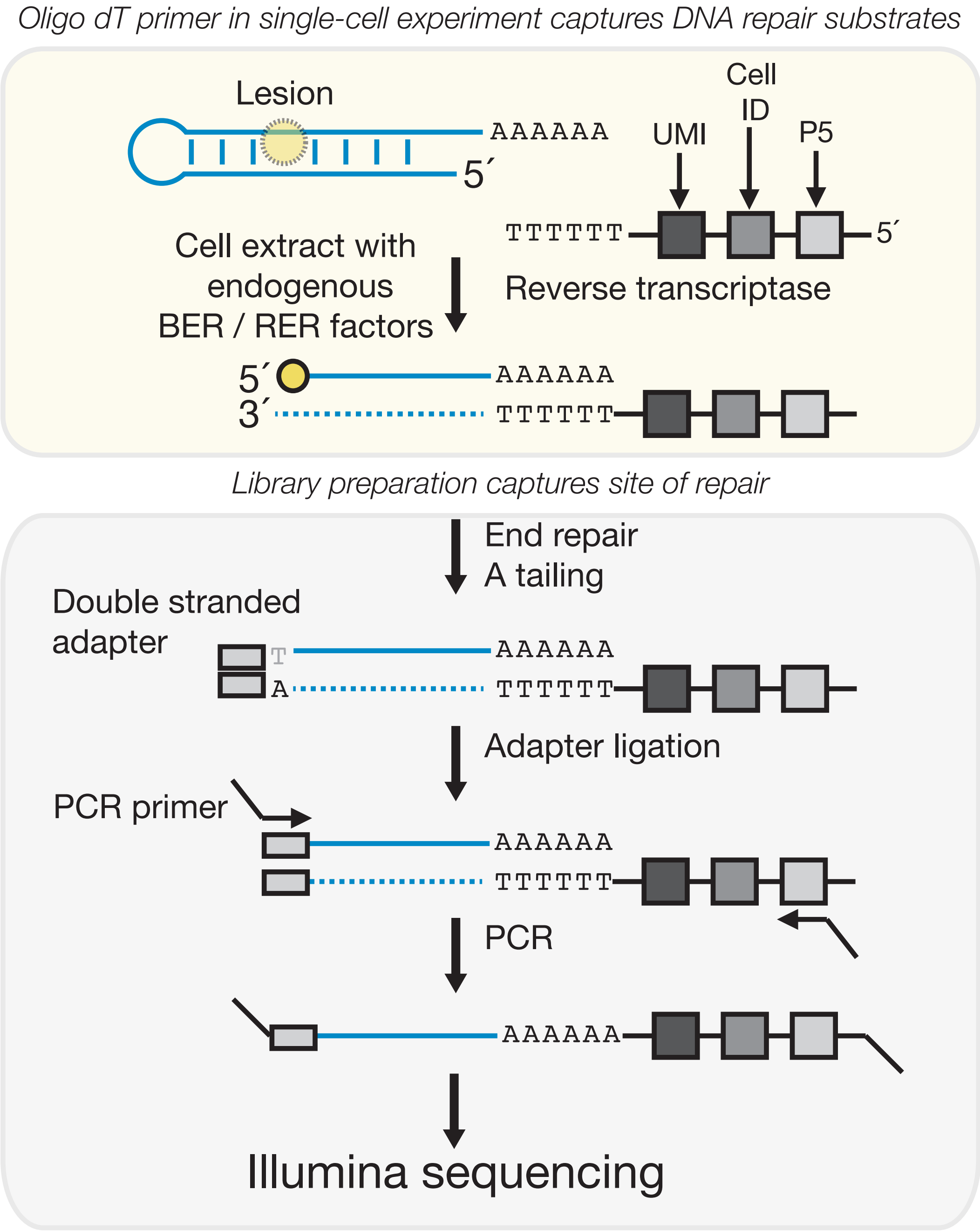

Supplementary Figure 2
Richer et al.

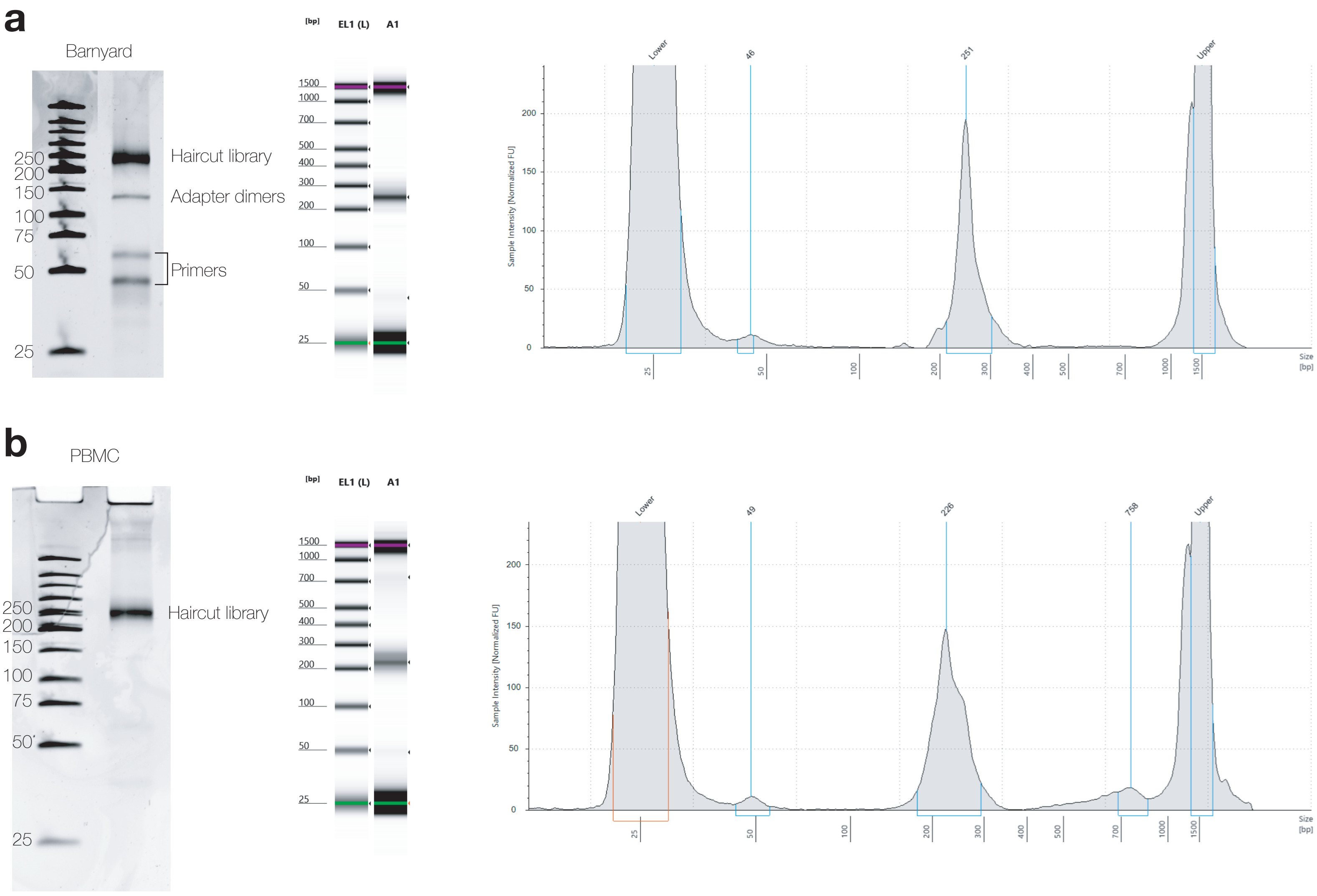

Supplementary Figure 3
Richer et al.

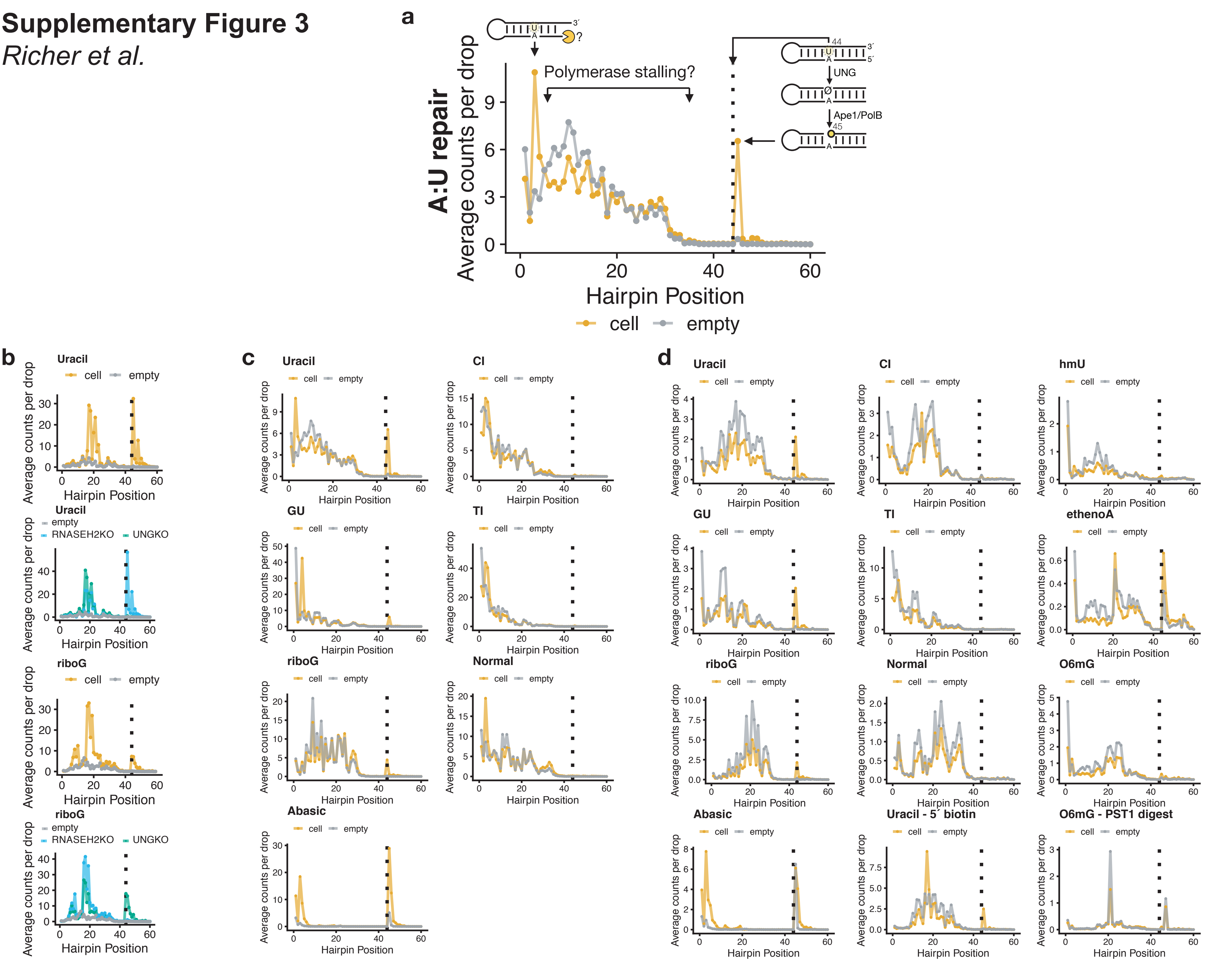

Supplementary Figure 4

Richer et al.

a

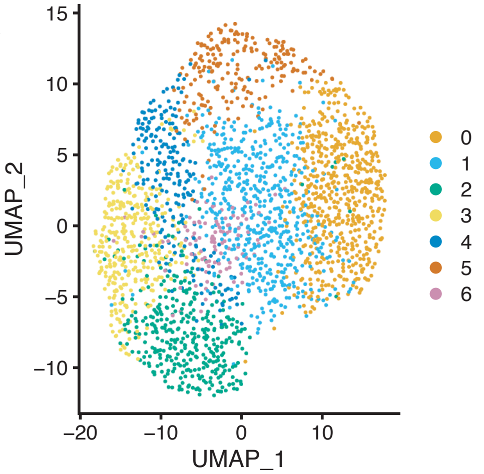

b

Cluster	UNG expression	RNASEH2C expression	Uracil repair	Ribonucleotide repair	Total number of cells
0	111	295	575	238	576
1	63	237	546	213	552
2	9	154	268	350	362
3	10	132	176	333	348
4	3	123	141	221	229
5	32	126	192	81	206
6	1	8	65	18	104
**Total**	**229**	**1075**	**1963**	**1454**	**2377**

c

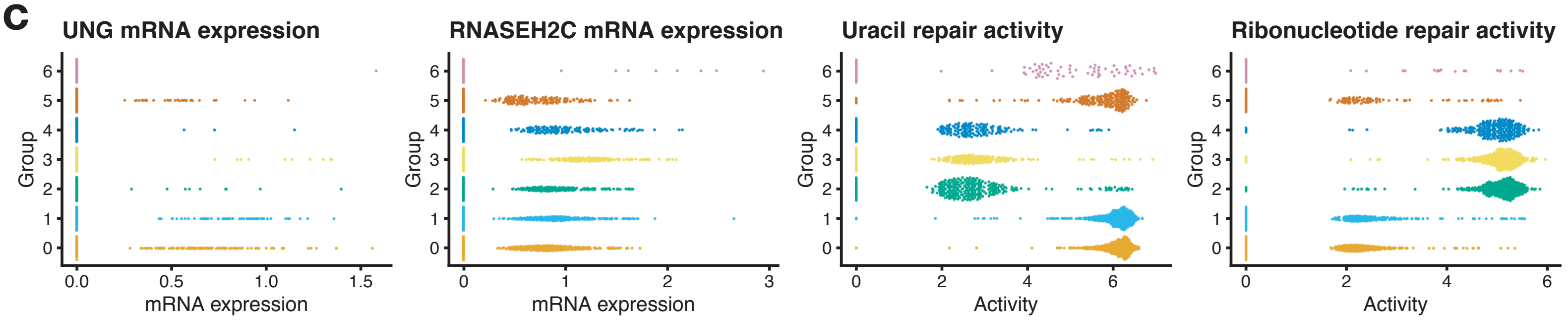

d

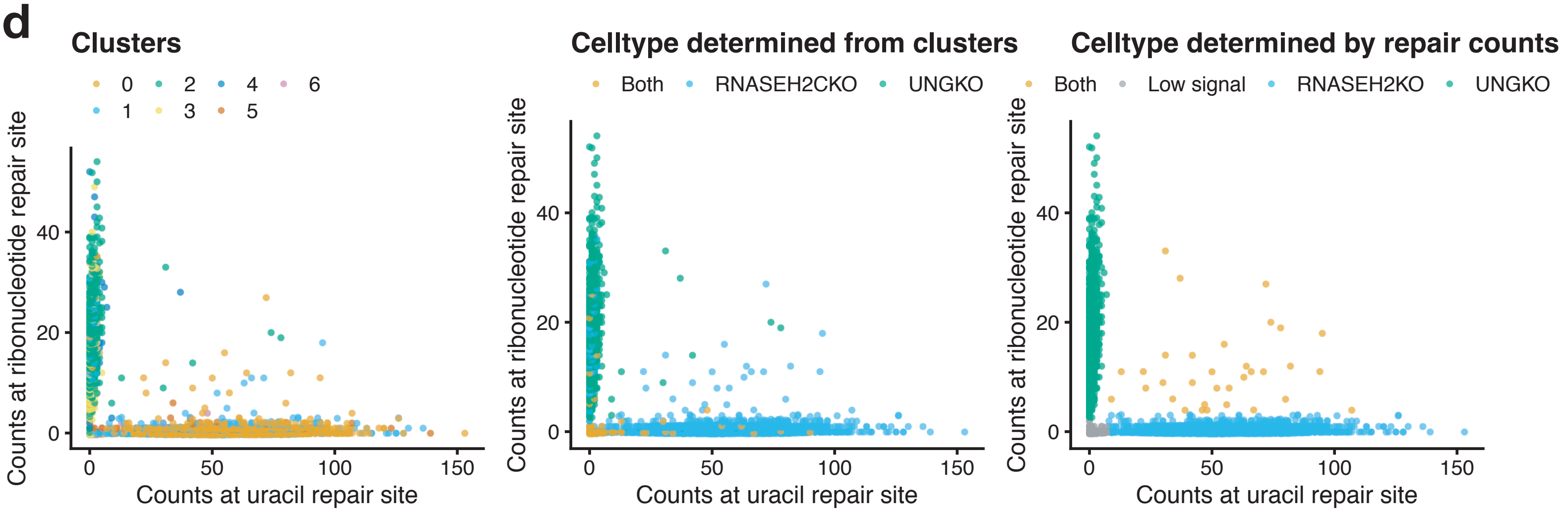

e

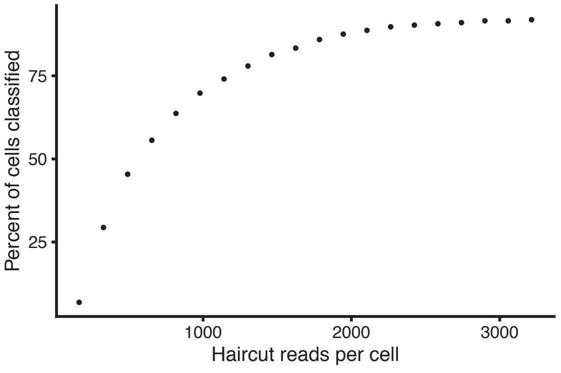

Supplementary Figure 5

Richer et al.

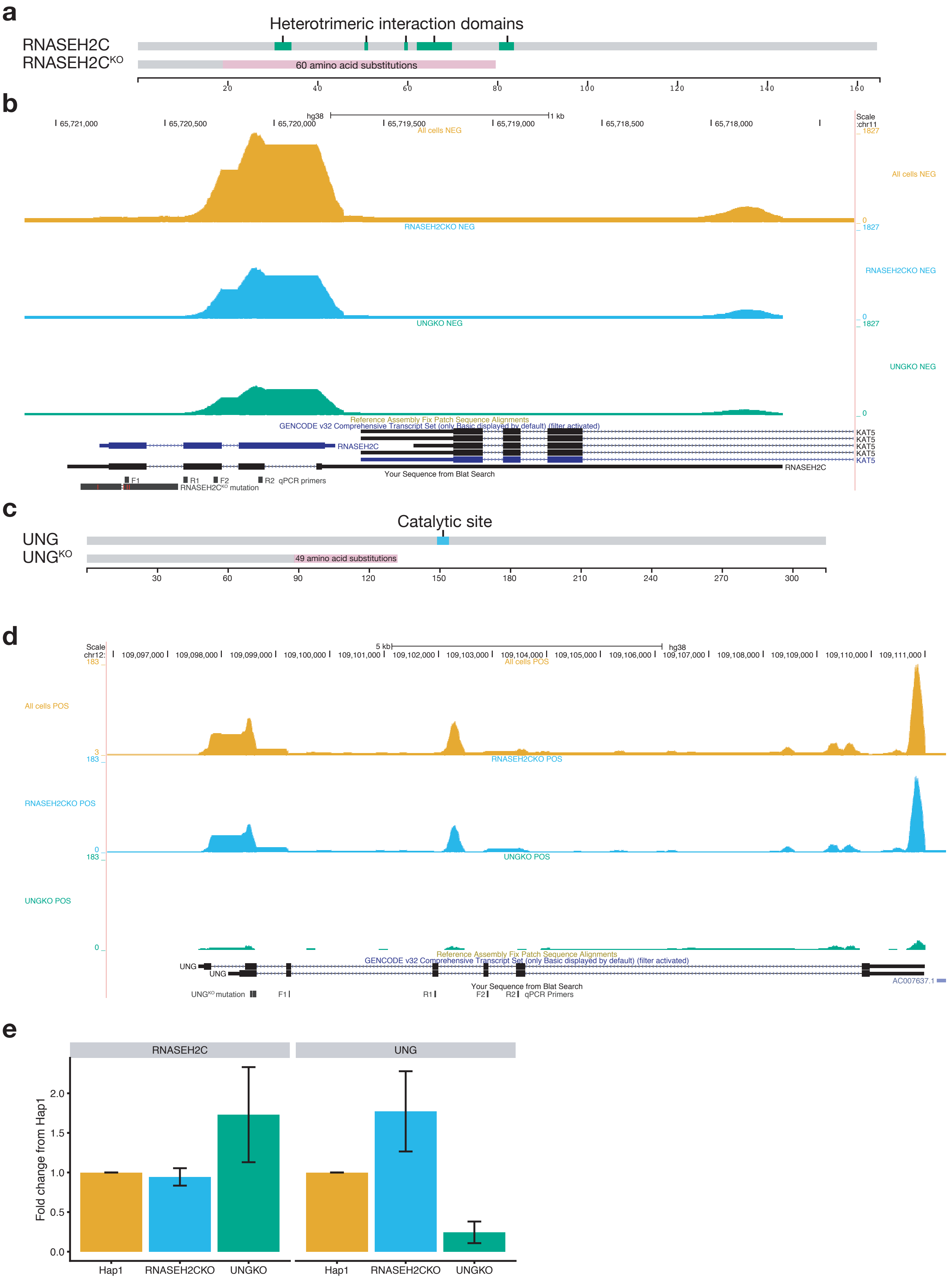

Supplementary Figure 6

Richer et al.

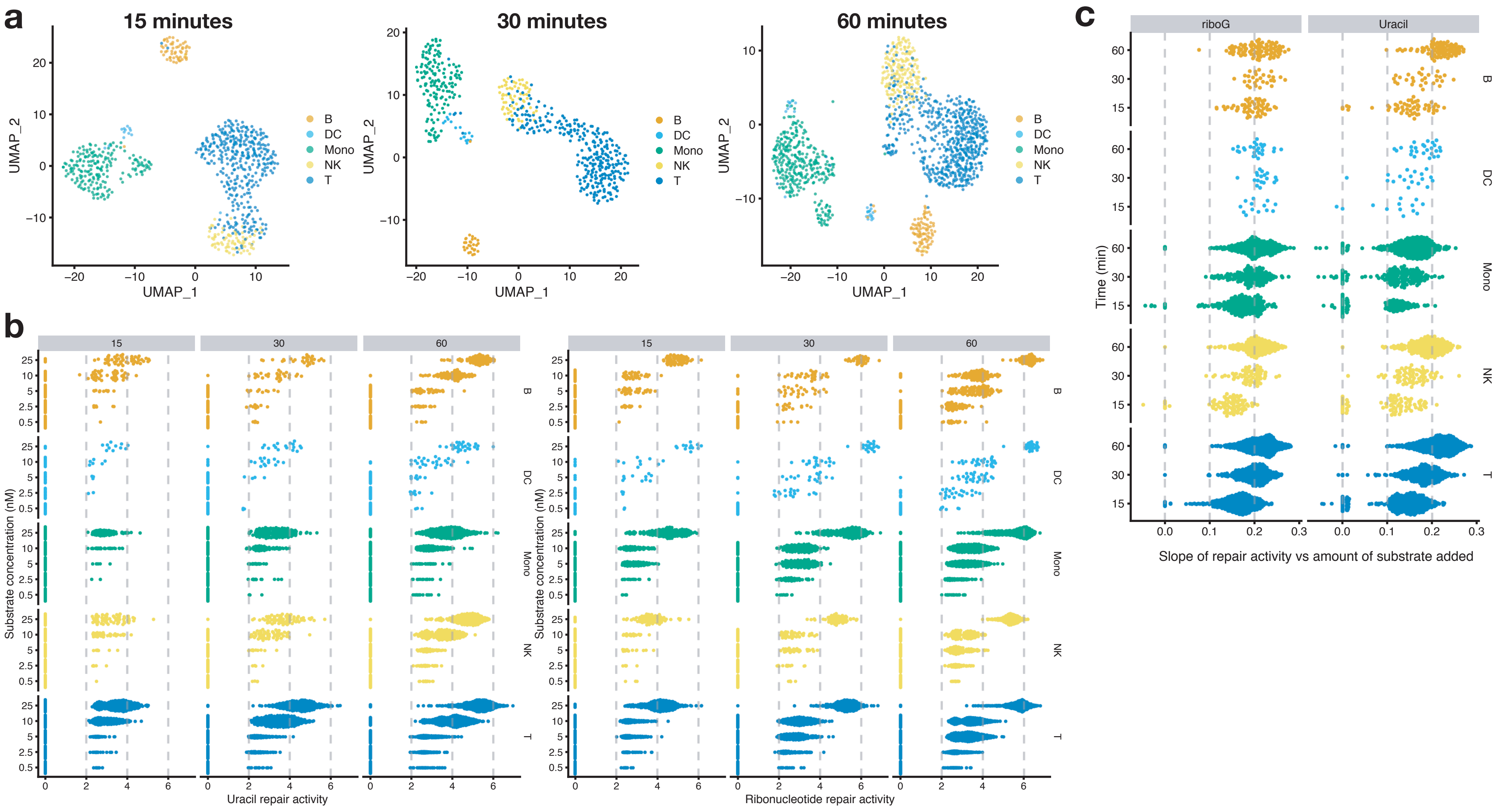

Supplementary Figure 7

Richer et al.

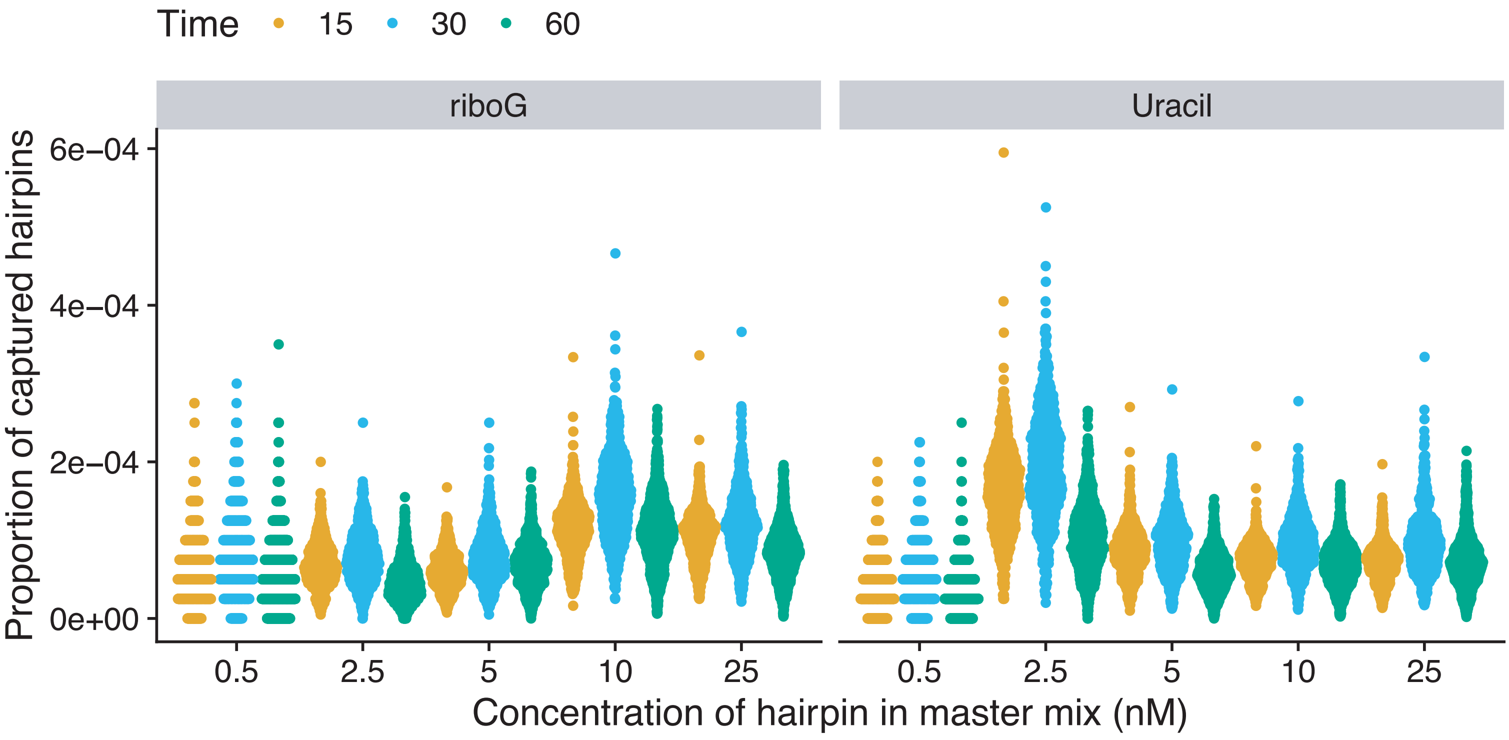

Supplementary Figure 8

Richer et al.

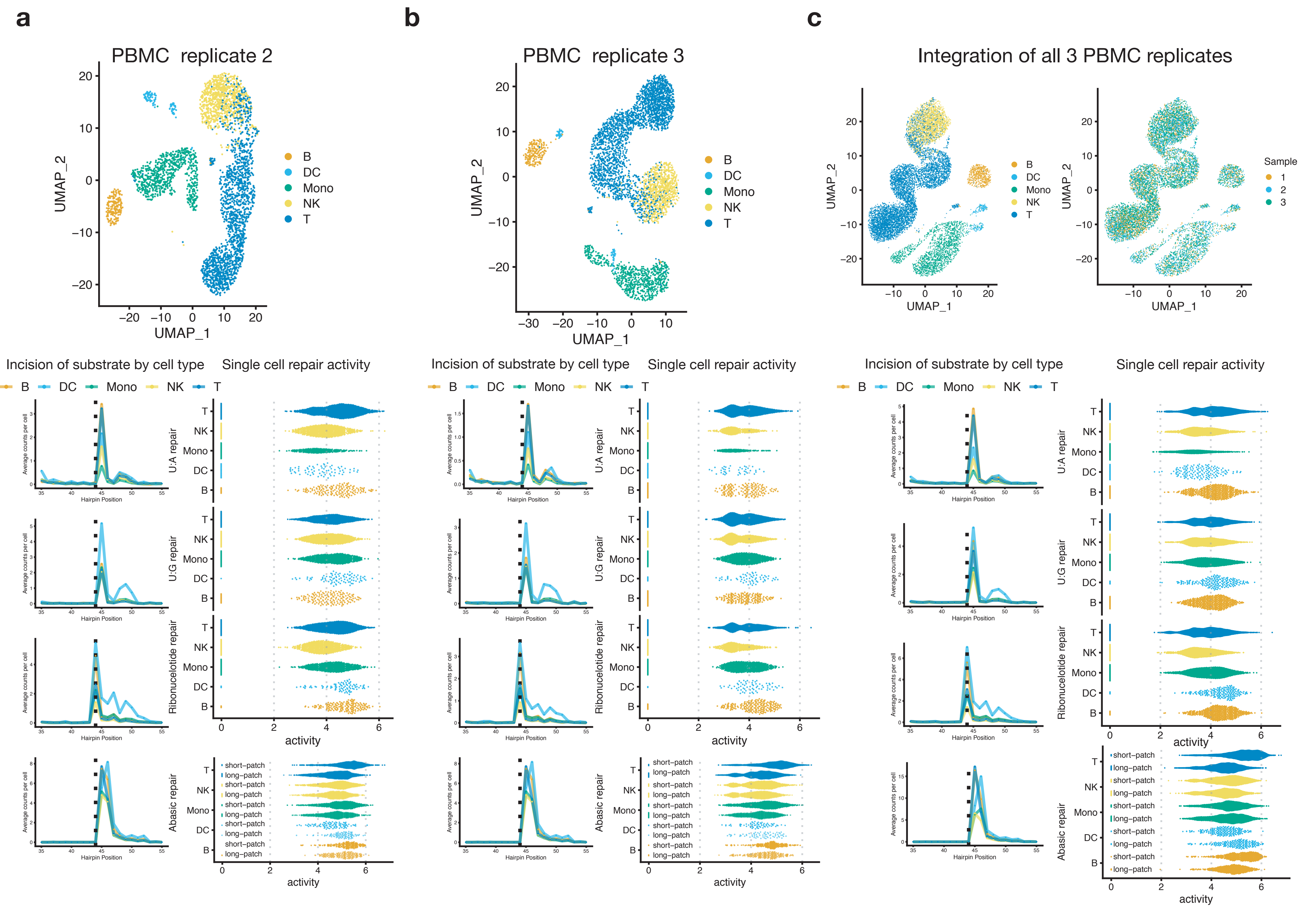

Supplementary Figure 9

Richer et al.

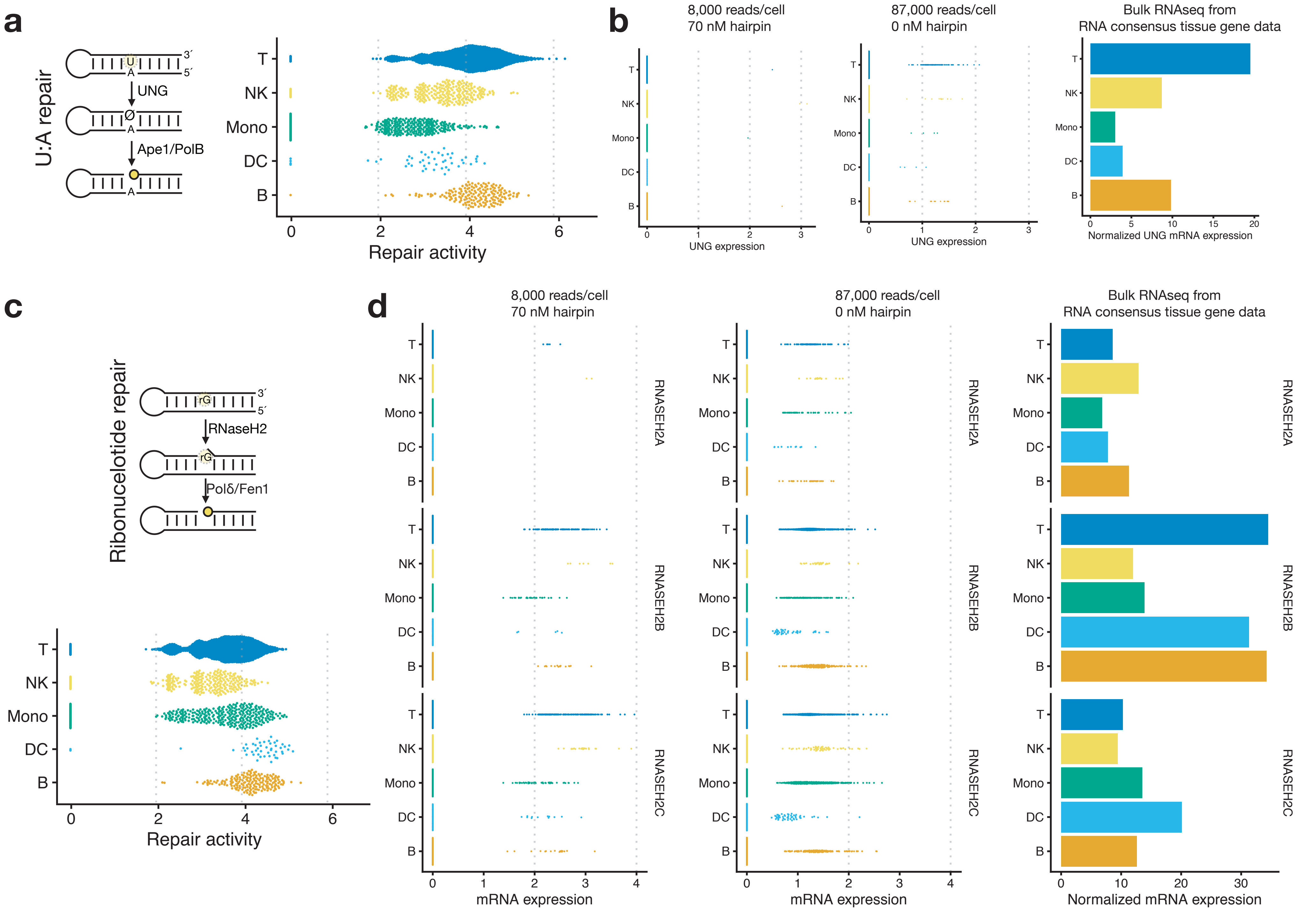

Supplementary Figure 10

Richer et al.

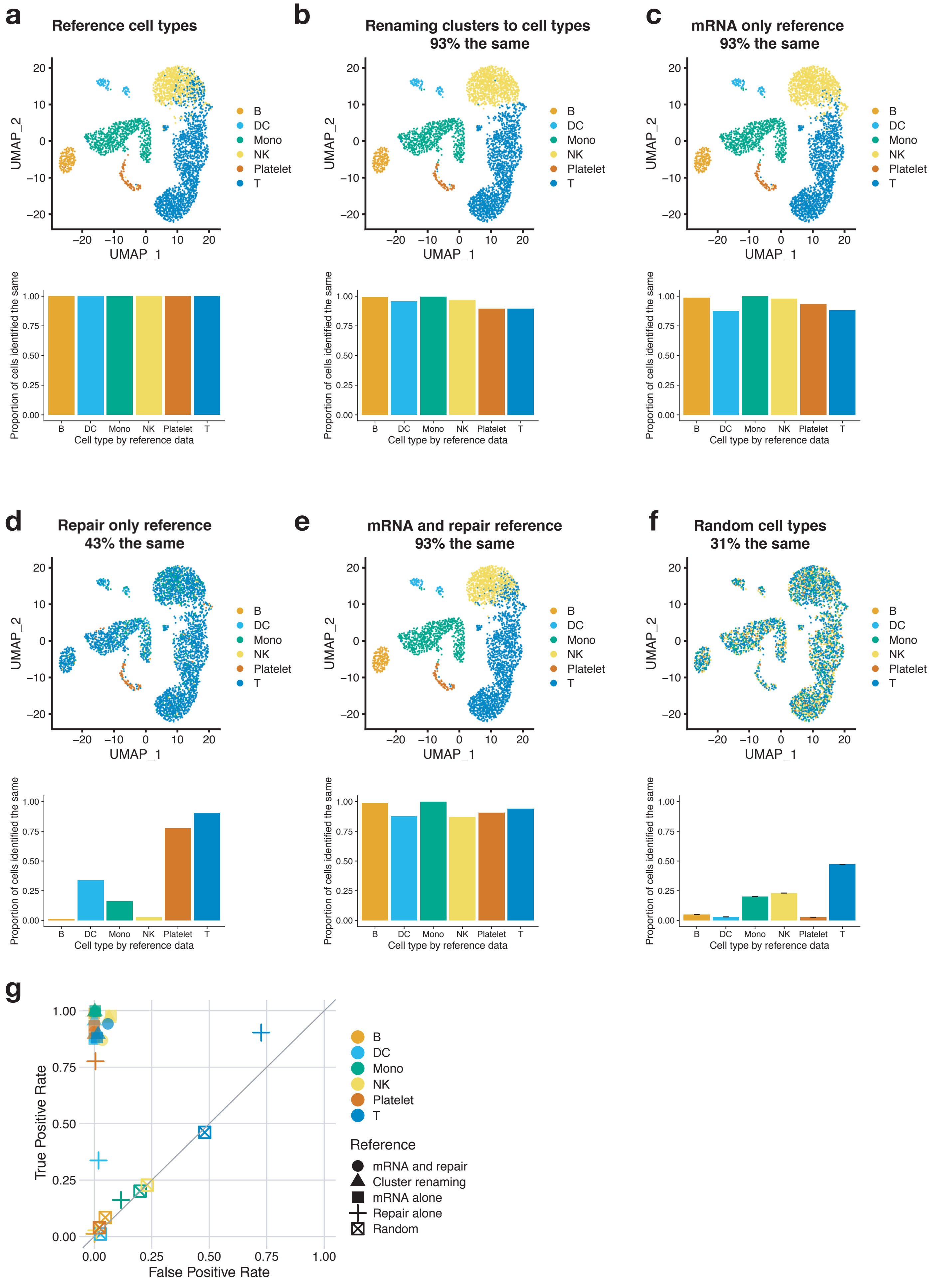

Supplement: gkaa240_Supplemental_Files [file gkaa240_supplemental_files.zip › Supplemetary Figures revised.pdf]
